# Coenzyme A metabolism: a key driver of gut microbiota dynamics and metabolic profiles

**DOI:** 10.1093/femsre/fuaf051

**Published:** 2025-10-11

**Authors:** Johanna Böttcher, Ody C M Sibon, Sahar El Aidy

**Affiliations:** Department of Microbiome Engineering, Swammerdam Institute for Life Sciences, University of Amsterdam, Science Park 904, 1098 XH Amsterdam, The Netherlands; Department of Biomedical Sciences; European Research Institute for the Biology of Ageing, University Medical Centre Groningen, 9713 AV Groningen, The Netherlands; Department of Microbiome Engineering, Swammerdam Institute for Life Sciences, University of Amsterdam, Science Park 904, 1098 XH Amsterdam, The Netherlands; Amsterdam Microbiome Expert Centre, University of Amsterdam, Science Park 904, 1098 XH Amsterdam, The Netherlands

**Keywords:** bacteria, Coenzyme A, metabolic diseases, metabolism, microbiota, pantothenate

## Abstract

Coenzyme A (CoA) biosynthesis is a crucial process in living organisms, characterized by the production of conserved intermediates through enzyme-catalysed steps that vary across species. The synthesis of CoA entails several conversions, starting from pantothenate. Pantothenate is an essential vitamin in humans and is synthesized by certain bacterial species. Intermediates of the biosynthetic pathway have been shown to impact bacteria, especially in community settings such as the intestinal microbiota. Additionally, various diseases have been associated with specific CoA precursors and metabolic pathways downstream of CoA in the gut microbiota, underscoring the significance of evaluating the current knowledge on how the CoA pathway influences the metabolic state of bacteria. This also highlights the importance of having standardized methodologies that can be employed to better understand the metabolism of the microbiome. In this review, we explore the current literature on bacterial CoA metabolism, with a particular focus on gut bacteria and the impact of CoA-related metabolites on bacterial composition, function and metabolism. Furthermore, we discuss previous and current methodologies employed to investigate CoA biosynthesis. Our goal is to provide valuable insights into the intricate relationship between CoA metabolism, gut microbiota and their implications for health and disease, offering a foundation for future research and therapeutic approaches.

## Teaser

This review evaluates bacterial Coenzyme A metabolism, with a focus on gut microbiota, the impact of CoA intermediates on bacterial function and composition, and their implications for gut health, alongside methodologies to study their role in microbial communities. Created in BioRender. Boettcher, J. (2025) https://BioRender.com/e85g138

## Introduction

Coenzyme A (CoA) is an essential cofactor in various biological processes across all living organisms (Leonardi et al. 2005). Its biosynthesis is therefore conserved among organisms, including humans and bacteria. However, variation exists in the expression and type of enzymes involved in CoA biosynthesis between bacteria and humans (Leonardi and Jackowski 2007). Vitamin B5 (pantothenate), the precursor of CoA, can be synthesized by certain bacteria, while others acquire it through transporters. In humans, pantothenate is obtained from dietary sources or synthesized by gut bacteria (Leonardi and Jackowski 2007, Uebanso et al. 2020). While the CoA biosynthesis pathway was discovered and studied decades ago, the impact of CoA intermediates on bacterial communities such as the human gut microbiota remains poorly understood. Recent evidence suggests that metabolic aberrations linked to CoA and related pathways may be associated with various metabolic and microbiota-related diseases, including inflammatory bowel disease (IBD), Parkinson’s disease (PD), type 2 diabetes mellitus (T2D), and other cardiovascular metabolic disorders (Qin et al. 2012, Anandhan et al. 2017, Jie et al. 2017, Guan 2019). Therefore, it is crucial to expand our understanding of how bacteria metabolize CoA intermediates and how the enzymes involved are regulated. A more detailed understanding of this pathway at the species-level also offers the potential to engineer and modify the pathway, which could deepen our insights into bacterial interactions and provide opportunities for interventions in disease states that are aimed to manipulate CoA metabolism in the gut microbiota.

### CoA biosynthesis and its regulation in gut bacteria

**Figure 1.**
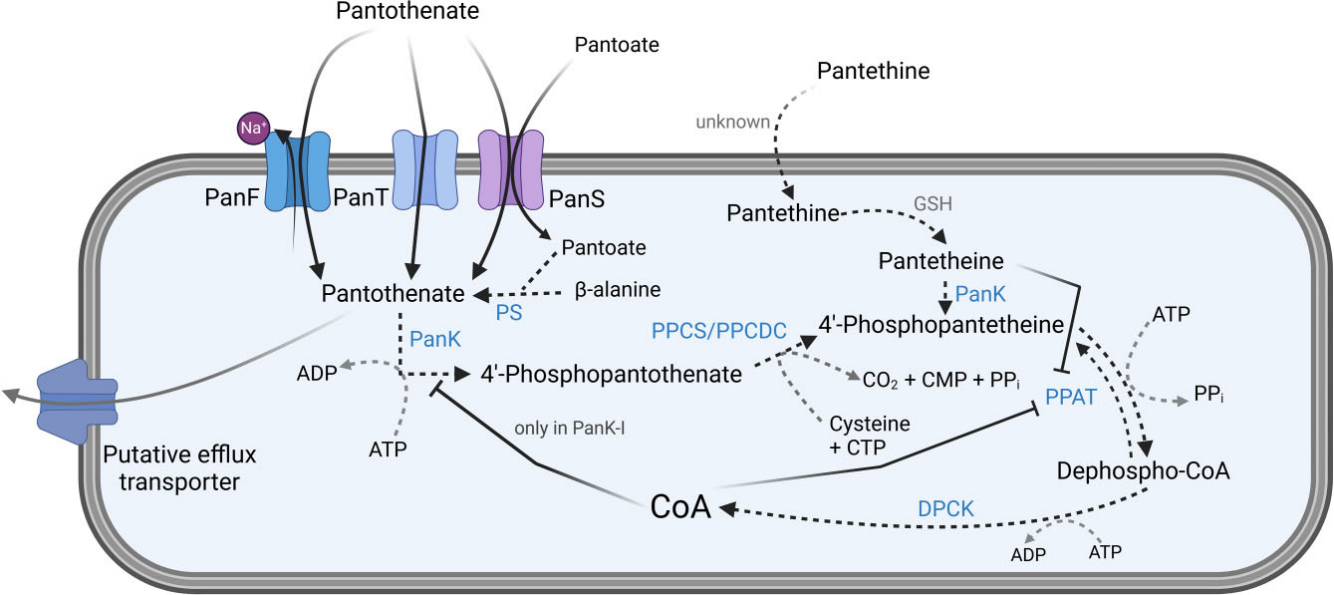
Summary of Coenzyme A biosynthesis in bacteria, including identified transporters. Pantothenate is transported into bacterial cells via three identified transporters -PanF, PanT, and PanS- and may be excreted through an efflux transporter. Inside the cell, pantothenate is converted into 4′-phosphopantothenate by pantothenate kinase (PanK), which exists as Type I, II, or III depending on the bacterial species. PanK-I is feedback-regulated by CoA. The biosynthetic pathway continues with the condensation of 4′-phosphopantothenate and cysteine to form 4′-phosphopantetheine via PPCS/PPCDC. This is followed by the addition of adenine monophosphate (AMP) to produce dephospho-CoA, a reaction catalysed by PPAT. The final step is the phosphorylation of dephospho-CoA by dephospho-CoA kinase (DPCK), yielding CoA, which can allosterically regulate PanK-I. Pantethine can enter bacterial cells through an unknown mechanism, where it is reduced to pantetheine and integrates into CoA metabolism via conversion to 4′-phosphopantetheine, a reaction catalysed by PanK-I. Both CoA and pantetheine act as inhibitors of PPAT. Created in BioRender, Boettcher (2025). Abbreviations: DPCK, dephospho-CoA kinase; GSH, glutathione; PanF, sodium/pantothenate symporter; PanK, pantothenate kinase; PanT, pantothenate transporter; PanS, pantothenate permease; PPAT, ATP:4′-phosphopantetheine adenylyltransferase; PPCS/PPCDC, 4′-phosphopantothenoylcysteine synthetase/4′-phosphopantothenoylcysteine decarboxylase; and PS, pantothenate synthetase.

CoA metabolism in the majority of bacteria, as in mammalian cells, relies on vitamin B5 (pantothenate) (Vallari and Jackowski 1988, Leonardi and Jackowski 2007). In the human gut, pantothenate is readily available, ingested normally through different dietary products such as meat, milk, eggs, legumes, and nuts (van den Berg 1997, Chungchunlam and Moughan 2024).Pantothenate can also be bound to CoA or 4′-phosphopantetheine in our food and as such it can be hydrolyzed via pantetheine to pantothenate by phosphatases and pantetheinases, respectively (Shibata et al. 1983). Free pantothenic acid is absorbed mainly in the small intestine via sodium-dependent multivitamin transporters with a bioavailability of around 50% (Tarr et al. 1981, Prasad et al. 1999, Said 2013, Walimbe et al. 2025). In contrast, bacterial pantothenate is produced in the large intestine and can be taken up by both human colonocytes and bacteria themselves; alternatively bacteria may synthesize it internally, depending on the expressed enzymes (Said et al. 1998, Said 2011). Typically, genes for either pantothenate synthesis or transport are present, but in some cases, such as in *Escherichia coli*, both can be part of the genome (Spry et al. 2008). Pantothenate can be transported into bacterial cells via the pantothenate permease PanF (encoded by the *panF* gene), first described in *E. coli* (Fig. 1).

Pantothenate excretion appears to occur via a separate efflux mechanism, as inactivation of *panF* did not affect the concentration of secreted pantothenate (Gerdes et al. 2002, Leonardi and Jackowski 2007). Bacteria of the phylum *Bacillota*, including *Lactobacillus* and some *Streptococci*, require a transporter for the uptake of extracellular pantothenate because they lack enzymes necessary for pantothenate synthesis (Rodionov et al. 2009). When pantothenate synthesis genes are present, the process begins with pantothenate synthetase (EC 6.3.2.1), which catalyses the condensation of β-alanine and pantoate, derived from L-aspartate and α-ketoisovalerate, respectively (Leonardi and Jackowski 2007). Once pantothenate is either synthesized or imported, the first step of the CoA biosynthetic pathway is initiated (Butman et al. 2020).

**Figure 2.**
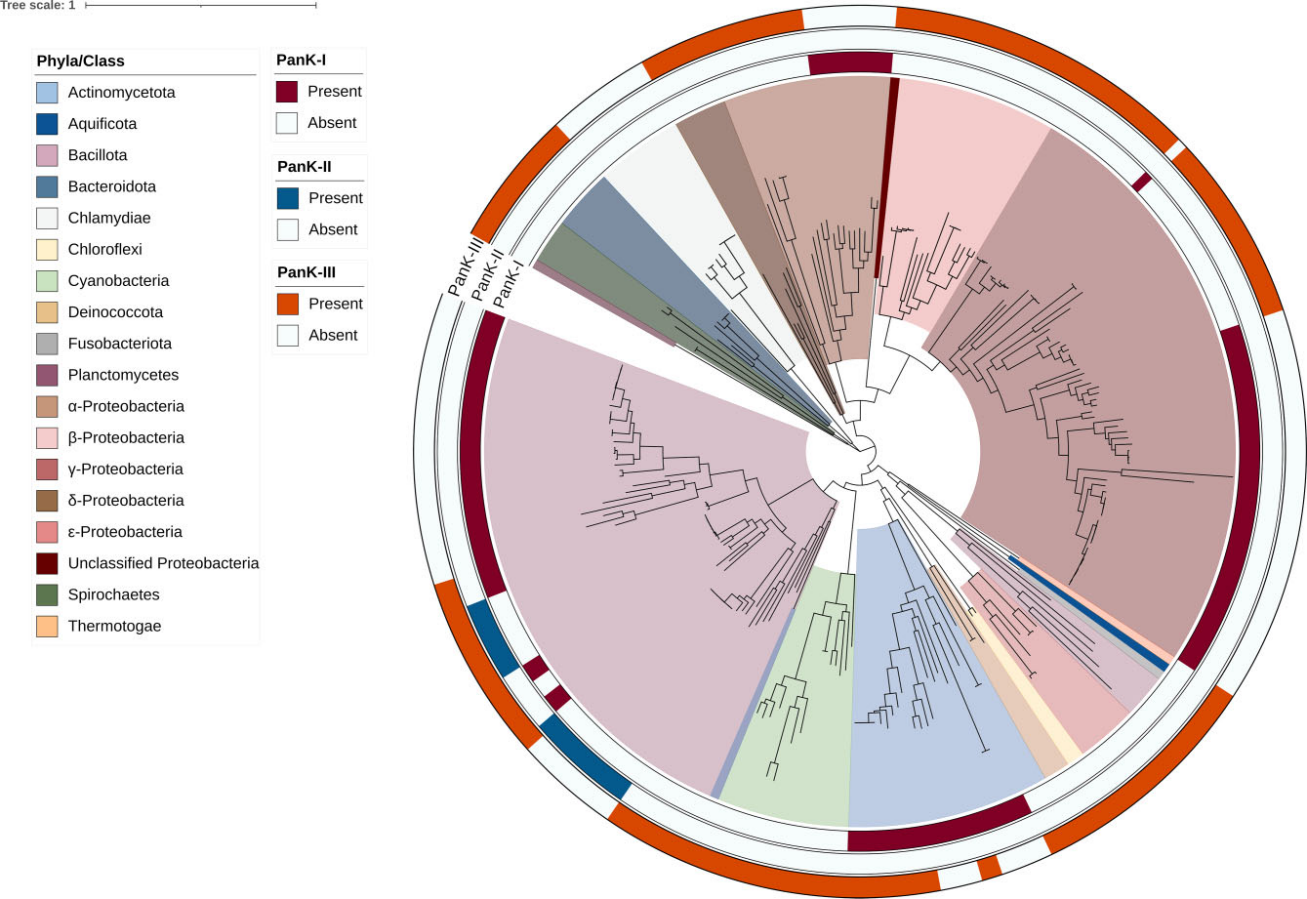
Phylogenetic tree of species investigated for pantothenate kinase types. The phylogenetic tree depicts the relationships between species analysed for the presence of pantothenate kinase (PanK) Types I, II, and III, based on findings by Yang et al. (2006). Species with PanK-I are represented in the inner-colored ring, PanK-II in the middle-colored ring , and PanK-III in the outer-colored ring. The phyla of the species are indicated in the figure legend with their respective colors, while for *Pseudomonadota*, the classes are shown instead of the phyla (α-, β-, γ-, δ-, ε-*Proteobacteria*).The phylogenetic tree was constructed using GTDB-Tk (Chaumeil et al. 2019) and available sequences listed by Yang et al. (2006) were downloaded for the alignment from NCBI.

Pantothenate kinase (PanK, EC 2.7.1.33), a key enzyme in the first step of CoA biosynthesis, phosphorylates pantothenate to generate 4′-phosphopantothenate (Fig. 1) (Vallari and Rock 1987). In bacteria, three PanK types have been identified: Type I, initially characterized in *E. coli* and often referred to as prokaryotic PanK (Leonardi and Jackowski 2007), Type II, identified in *Staphylococcus aureus*, which shares genetic similarities to the eukaryotic PanK (Leonardi et al. 2005), and Type III, initially found in pathogenic bacteria, including *Pseudomonas aeruginosa, Bordetella pertussis*, and *Helicobacter pylori* (Brand and Strauss 2005, Yang et al. 2006). Yang et al. (2006) investigated the phylogenetic distribution of all three PanK types and found that PanK-III is present in almost all bacterial groups analysed, including *Actinomycetota, Bacteroidota, Bacillota*, and *Pseudomonadota* with the exception of *Chlamydiae*, which lack all three PanK types (Yang et al. 2006). In contrast, PanK-I was found only in *Actinomycetota, Bacillota* and *α-* and *γ-Proteobacteria*, while PanK-II was restricted to *Bacillota* (Fig. 2) (Yang et al. 2006). This suggests that PanK-III is likely the most prevalent pantothenate kinase in gut bacteria, though considerable variability exists between bacterial taxa.

The conversion of pantothenate to 4′-phosphopantothenate via PanK is allosterically regulated through feedback inhibition by CoA and its thioesters. Once CoA and its thioesters are produced, they can compete with adenine triphosphate (ATP) for binding to PanK (Fig. 1). However, this regulation has only been observed in PanK-I, as PanK-II and PanK-III lack this mechanism (Vallari et al. 1987, Rock et al. 2003, Leonardi et al. 2005, Shimosaka et al. 2016). Notably, CoA is a more potent inhibitor than its thioesters (Vallari et al. 1987). In *S. aureus*, PanK does not undergo feedback inhibition by CoA, as this species relies on a CoA/CoA disulfide reductase redox system that requires high CoA concentrations to detoxify reactive oxygen species, rather than glutathione (Leonardi et al. 2005). PanK-II is rarely expressed in bacteria compared to the other two types and is thought to have been horizontally transferred from eukaryotes, which may explain the similarity between the mammalian and bacterial enzyme (Choudhry et al. 2003). This suggests that different regulation mechanisms control the conversion of pantothenate to 4′-phosphopantothenate, with the impact of CoA and its thioesters varying between bacterial species.

Once 4′-phosphopantothenate is produced, it is condensed with cysteine to form 4′-phosphopantothenoylcysteine, which is then decarboxylated to 4′-phosphopantetheine (Fig. 1). This conversion occurs with the help of cytidine triphosphate rather than ATP (Strauss et al. 2001). In most bacteria, this enzymatic conversion is catalysed by a fused gene (*coaBC*), which encodes a bifunctional enzyme with 4′-phosphopantothenoylcysteine synthetase and 4′-phosphopantothenoylcysteine decarboxylase activity (PPCS/PPCDC, EC 6.3.2.5). However, in *Enterococcus* and *Streptococcus*, two separate enzymes are responsible for these reactions, similar to eukaryotic organisms (Gerdes et al. 2002, Leonardi and Jackowski 2007, Balibar et al. 2011).

The next step in the CoA biosynthesis is catalysed by the ATP:4′phosphopantetheine adenylyl transferase (PPAT, EC 2.7.7.3), which adds an AMP to 4′-phosphopantetheine through ATP (Fig. 1). This was first identified in *Corynebacterium ammoniagenes* and *E. coli* (Martin and Drueckhammer 1993, Geerlof et al. 1999). The addition of AMP generates 3′-dephospho-CoA and pyrophosphate, though, this transfer is reversible and allosterically regulated through CoA (Izard 2003, Chatterjee et al. 2016). The regulation is dependent on the concentrations of ATP, 4′-phosphopantetheine and 3′-dephospho-CoA, as these compounds compete with CoA for PPAT binding (Miller et al. 2007). Yoon et al. (2011) elucidated the structure of PPAT in *E. faecalis* in different binding states using ATP and the active CoA moiety pantetheine. Their results demonstrated that pantetheine binds similarly to CoA, inhibiting PPAT activity (Yoon et al. 2011). The final step for CoA biosynthesis is the ATP-dependent phosphorylation of 3′-dephospho-CoA to CoA via the dephospho-CoA kinase (EC 2.7.1.24), which was also first identified in *C. ammoniagenes* and *E. coli* (Mishra et al. 2001).

To gain further insights into pantothenate biosynthesis in gut bacteria, Magnúsdóttir et al. (2015) systematically assessed gut bacterial genomes for pantothenate biosynthetic genes (Magnúsdóttir et al. 2015). Their analysis revealed that only few *Bacillota* and *Actinomycetota* genomes contain the necessary enzymes for pantothenate synthesis, while these genes are entirely absent in *Fusobacteriota*. In contrast, the genomes of *Bacteroidota* and *Pseudomonadota* were predicted to contain all the enzymes required for pantothenate biosynthesis (Magnúsdóttir et al. 2015). When biosynthetic genes are absent, bacteria rely on transport mechanisms to acquire pantothenate from external sources. In addition to PanF, a transporter called PanS was identified in *Salmonella enterica*, which transports not only pantothenate but also its precursors, ketopantoate and pantoate (Ernst and Downs 2015). Moreover, the pantothenate transporter PanT was found in *Levilactobacillus brevis*, which shares an energy-coupling factor module with transporters of folate and hydroxymethylpyrimidine (Zhang et al. 2014). Subsequently, *panS* orthologs were found in various orders of the *Bacillota* phylum, including *Eubacteriales, Veillonellales*, and *Tissierellales* (Rodionov et al. 2019).

Following CoA biosynthesis, this reactive cofactor is rapidly converted into acetyl-CoA and acyl-CoA, central intermediates in metabolic processes such as the tricarboxylic acid (TCA) cycle, fatty acid pathways, and short-chain fatty acid (SCFA) production (Flint et al. 2015, Krivoruchko et al. 2015). Acetyl-CoA is produced either from CoA and acetate, a SCFA that is commonly produced by gut bacteria from dietary fibre (Hosmer et al. 2023), or via the degradation of fatty acids, which can also yield propionyl-CoA. Acetyl-CoA then fuels the TCA cycle, supports fatty acid biosynthesis and contributes to SCFA production (Flint et al. 2015, Krivoruchko et al. 2015). Furthermore, CoA can be recycled via the reduction to 4′-phosphopantetheine, which can be transferred to the acyl carrier protein apoprotein (apo-ACP) resulting in holo-ACP (Elovson and Vagelos 1968, Lambalot and Walsh 1995). Holo-ACP is essential for the acyl-group activation during fatty acid biosynthesis (Lambalot and Walsh 1995). Together, these processes illustrate the intricate interplay of different CoA-containing metabolites in essential metabolic pathways in bacteria.

In conclusion, pantothenate biosynthesis varies across bacterial species, including those inhabiting the human gastrointestinal tract, while CoA synthesis remains a conserved pathway. CoA can then react with essential metabolic intermediates that are intertwined in central pathways, underscoring the essential role of CoA in bacterial metabolism and cellular function.

### The role of CoA intermediates in shaping gut microbial ecology

Intermediates of the CoA biosynthesis pathway have the potential to influence bacterial communities, with particular implications for those within the gut microbiome. This section evaluates key intermediates that may impact gut bacteria, drawing on relevant literature to better understand their role in shaping microbial dynamics and community composition in the gut.

### Role of pantothenate in bacterial growth and cross-feeding

Pantothenate is a crucial intermediate in the early stages of CoA biosynthesis and is an essential vitamin for both humans and bacteria. In the large intestine, most dietary pantothenate has been absorbed by the human host. This leaves the bacteria to produce and share their own vitamin B5, in competition with colonocyte absorption (Said 2013, Magnúsdóttir et al. 2015). Most bacteria can synthesize pantothenate from β-alanine and pantoate (Fig. 1) (Tahiliani and Beinlich 1991), with genomic predictions indicating that all *Bacteroidota*, most of the *Pseudomonadota* and some of *Actinomycetota* and *Bacillota* genomes contain the pantothenate biosynthetic pathway (Magnúsdóttir et al. 2015). This, however, still leaves a large proportion of the gut microbiota that relies on the cross-feeding of pantothenate in the colon (Sharma et al. 2019, Uebanso et al. 2020, Culp and Goodman 2023).

Presumably, differences in pantothenate metabolism occur in the small and the large intestine. While the small intestine is consistently inhabited by *Streptococcus* and *Veillonella*, which belong to the *Bacillota* phyla, the abundance of *Clostridium* and *Escherichia* can vary (Booijink et al. 2010, El Aidy et al. 2015). Furthermore, the upper small intestine contains more *Pseudomonadota, Actinomycetota, Fusobacteriota*, and overall more *Bacillota* compared to stool samples (Leite et al. 2020). Accordingly, since only some *Bacillota* genomes contain a full biosynthetic pathway for pantothenate (Magnúsdóttir et al. 2015), species more predominant in the small intestine and therefore in higher contact with dietary pantothenate might generally express less biosynthetic genes. However, a clear distinction cannot be concluded yet since taxa connected with pantothenate biosynthetic genes are also present. The large intestine, which contains overall more bacteria, consists predominantly of the phyla *Bacillota, Bacteroidota*, and *Pseudomonadota* with *Bacteroidaceae, Lachnospiraceae*, and *Oscillospiraceae* as predominant families, which are taxa known to have pantothenate biosynthetic genes (Chinda et al. 2022, Jiao et al. 2022). Therefore, the localization of the bacteria in the gut does not necessarily determine their need to express pantothenate biosynthetic genes but rather emphasizes the importance of cross-feeding between gut bacterial species (Magnúsdóttir et al. 2015).

The abundance of genes responsible for pantothenate biosynthesis varies across different metagenomes, depending on the geographical region. For example, fecal samples from a Chinese cohort were enriched in pantothenate biosynthetic genes, while samples from a US cohort have fewer such genes. This difference was hypothesized to reflect the influence of a Western diet on the microbial community (Tarracchini et al. 2024). Four bacterial species -*Agathobacter rectalis, Bacteroides spp., Phocaeicola dorei*, and *Segatella copri-* were predicted to play a key role in B-vitamin biosynthesis (Tarracchini et al. 2024).

Notably, some bacteria, such as *A. rectalis, Anaerobutyricum hallii, Anaerostipes caccae, Anaerostipes hadrus*, and *Roseburia intestinalis* are capable of growing without extracellular pantothenate when their biosynthetic genes are expressed (Soto-Martin et al. 2020). In contrast, species such as *Lactobacillus helveticus, Faecalibacterium prausnitzii, Subdoligranulum variabile, Coprococcus catus*, and *R. inulinivorans*, require pantothenate for growth, as demonstrated in *in vitro* assays with pantothenate-depleted medium (Yao et al. 2018, Soto-Martin et al. 2020). In *L. helveticus*, pantothenate deficiency resulted in a decreased activity of enzymes related to glucose, biotin, and fatty acid metabolism as well as fatty acid synthesis, and alongside an upregulation of genes encoding ATP-dependent transporters (Yao et al. 2018). Furthermore, depletion of pantothenate in *E. faecalis* led to a decrease in the intracellular CoA pool and overall growth. CoA was turned over into ACP, which depleted the CoA pool, possibly affecting downstream metabolic pathways relating to growth (Das and Toennies 1969).

Next to the depletion of growth when pantothenate is lacking, increasing pantothenate concentrations led to the increase of *E. coli* growth, which was shown to be proportional to the increase in internal CoA content (Powell et al. 1969). Similarly, when *E. faecalis* was supplemented with different pantothenate concentrations, low concentrations led to normal exponential growth with an initial proportional increase in CoA, which reached its maximum before the growth and gradually decreased after, while high pantothenate led to maximum CoA increase and it remained constant during exponential growth (Toennies et al. 1966).

These findings highlight the essential role of pantothenate in supporting growth for certain gut bacteria, which must either synthesize or uptake the vitamin, an adaptation reflected in the upregulation of synthesis genes. Moreover, pantothenate directly influences the intracellular CoA pool, potentially shaping overall cell growth and metabolism.

### 4′-Phosphopantetheine: an intermediate with limited uptake mechanisms

4′-Phosphopantetheine, the second intermediate in the CoA biosynthesis and precursor of ACP, has been primarily studied in *E. coli* and is produced through two primary processes: the CoA biosynthetic pathway and the degradation of acyl carrier protein (ACP). Unlike pantothenate, 4′-phosphopantetheine lacks known uptake mechanisms in *E. coli* (Vallari and Jackowski 1988). This limitation also applies for other phosphorylated intermediates of the CoA biosynthetic pathway, such as 4′-phosphopantothenate and 4′-phosphopantothenoylcysteine, which similarly lack uptake pathways (Jackowski and Rock 1984, Gerdes et al. 2002). Notably, Jackowski and Rock (1981, 1984) hypothesized the potential excretion of 4′-phosphopantetheine since supplementation of β-alanine in *E. coli* led to increased extracellular 4′-phosphopantetheine, which was later connected with ACP degradation (Jackowski and Rock 1981, 1984). Similarly, Yu et al. (2022) demonstrated that 4′-phosphopantetheine concentrations increased in the medium of *Lactococcus lactis* cultures when treated with pantethine, the active moiety of CoA (Yu et al. 2022). This indicates that external supplementation of 4′-phosphopantetheine precursors might lead to excess production and therefore excretion of 4′-phosphopantetheine.

Lastly, Jackowski and Rock (1984) showed that 4′-phosphopantetheine cannot be directly salvaged or reused by bacterial cells, highlighting its distinct metabolic handling compared to other CoA intermediates (Jackowski and Rock 1984). This raises intriguing questions about its potential role in interspecies interaction or signalling within bacterial communities.

### Pantethine and pantetheine as alternative routes to CoA biosynthesis in bacteria

Pantetheine, the reduced form of pantethine and active moiety of CoA, is an intermediate of the dietary scavenging of pantothenate from CoA and 4′-phosphopantetheine, which occurs primarily in the small intestine and only in very low amounts in the large intestine due to lower expression of pantetheinases (Millet et al. 2023, Chungchunlam and Moughan 2024). Its potential role in gut bacterial metabolism is scarcely explored and has mainly been evaluated as nutritional supplement for both bacteria and humans (Rana et al. 2010, Balibar et al. 2011, Rumberger et al. 2011).

**Figure 3.**
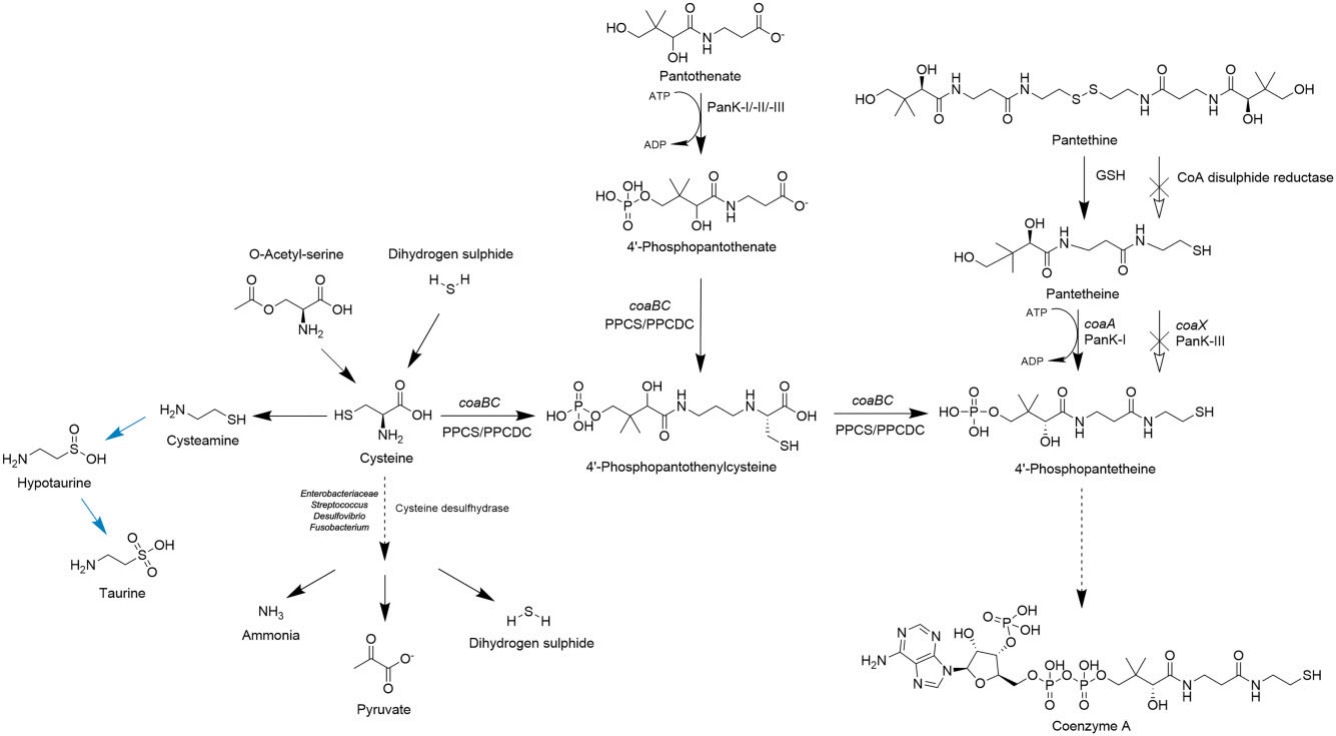
Intermediates and derivatives of the CoA biosynthetic pathway are intricately intertwined and regulated. Pantethine integrates into CoA metabolism via *coaA*. In bacteria, pantethine is reduced to pantetheine using glutathione as a detoxifier of reactive oxygen species, rather than CoA. Pantetheine is phosphorylated to 4′-phosphopantetheine in bacteria expressing *coaA* (PanK-I), a process absent in bacteria with *coaX* (PanK-III). The 4′-phosphopantetheine can be further converted into CoA, which participates in various metabolic pathways. Cysteine, a sulphur-containing amino acid, is synthesized from O-acetyl-serine and dihydrogen sulphide and may enter the CoA pathway via 4′-phosphopantothenylcysteine or be metabolized into ammonia, pyruvate, and dihydrogen sulphide, which serve downstream functions. Additionally, cysteine can be converted to cysteamine and, in mammals, to hypotaurine and ultimately taurine. Reactions specific to mammals are indicated with blue arrows. Created with ChemDraw. Abbreviations: GSH, glutathione; PanK, pantothenate kinase; and PPCS/PPCDC, 4′-phosphopantothenoylcysteine synthetase/4′-phosphopantothenoylcysteine decarboxylase.

Pantetheine can be oxidized to its stable form pantethine and reduced back via glutathione and glutathione reductase (EC 1.8.1.7) (Fig. 3) (Durr and Cortas 1964). However, this mechanism is absent in bacteria lacking the glutathione thiol/disulfide redox system, such as *S. aureus*. Instead, *S. aureus* employs a CoA/CoA disulphide redox system involving CoA disulphide reductase (EC 1.8.1.14), which allows it to reduce CoA disulphides and 4,4′-diphosphopantethine but not oxidized glutathione, cystine, pantethine, or hydrogen peroxide (Fig. 3) (delCardayre et al. 1998). Pantethine and pantetheine are not direct intermediates in the canonical CoA biosynthetic pathway but serve as alternative substrate for PanK-I, an enzyme expressed from *coaA* in *E. coli*. PanK-I can convert pantetheine to 4′-phosphopantetheine, bypassing the need for PPCS/PPCDC (*coaBC)* (Fig. 3) (Balibar et al. 2011). This alternative pathway enables survival in *S. enterica coaBC* mutants when pantethine is present (Ernst and Downs 2015). However, this reaction is species-specific; e.g. *P. aeruginosa* PanK-III lacks affinity to pantethine, rendering the pathway inoperative in this bacterium (Balibar et al. 2011).

PanK-I in *E. coli* phosphorylates both pantothenate and pantethine, with a higher affinity for pantethine. In experiments where *coaBC* expression was disrupted, pantethine but not pantothenate, CoA or dephospho-CoA rescued growth, underscoring the inability of upstream CoA intermediates to compensate for missing enzymes and the lacking uptake of CoA and dephospho-CoA (Balibar et al. 2011). Similarly, *coaD* (PPAT) mutants in *E. coli* could not be rescued by any metabolites, including pantethine, demonstrating the limitation of the alternative pathway to rescue deficiencies downstream of *coaBC* (Balibar et al. 2011).

To understand pantethine uptake, Balibar et al. (2011) knocked out the *panF* gene, which encodes the pantothenate transporter, and tested the viability of *E. coli* in the presence of pantethine. Pantethine restored growth in the *panF* mutant, indicating that its uptake is independent of *panF* (Balibar et al. 2011). Likewise, *S. enterica* lacking the *panS* transporter gene exhibited restored growth in the presence of pantethine, suggesting a PanF- and PanS-independent mechanism for pantethine uptake (Ernst and Downs 2015). Notably, Warneke et al. (2024) identified the uptake of a novel pantetheine derivative, cysteinopantetheine, via the cystine transporter TcyJKLMN in *Bacillus subtilis*, but not of pantetheine or pantothenate, suggesting an alternative uptake mechanism independent of pantothenate transporters (Warneke et al. 2024).

In summary, pantetheine and pantethine provide an alternative route to CoA biosynthesis in bacterial species with PanK-I, most likely occurring predominantly in the small intestine due to the presence of phosphatases producing the intermediates. These metabolites are taken up via an unknown mechanism independent of PanF and PanS but potentially as derivative such as cysteinopantetheine. Pantethine can rescue CoA biosynthesis when upstream enzymes are non-functional, but it cannot bypass deficiencies in downstream enzymes such as PPAT. This highlights their role as critical components of an alternative CoA biosynthetic pathway in bacteria capable of utilizing PanK-I.

### Cysteine and taurine: from CoA biosynthesis to microbial modulation

Cysteine, an essential sulphur-containing amino acid, is critical for the production of 4′-phosphopantetheine and plays an essential role in CoA synthesis. Additionally, it is vital for the biosynthesis of key vitamins such as biotin, and thiamine as well as other sulfur-containing compounds (Wada and Takagi 2006, Barton et al. 2017). Cysteine can be synthesized by bacteria or taken up from the colonic environment after dietary proteins are degraded (Blachier et al. 2019). Due to its reactive nature, cysteine concentrations are tightly regulated, as low levels have been shown to cause cytotoxicity (Sørensen and Pedersen 1991, Korshunov et al. 2020). Supporting this, free cysteine concentrations in the colon are generally low, reflecting its rapid and efficient metabolism (Ahlman et al. 1993).

Notably, the effect of cysteine concentrations on the balance of CoA metabolism in bacteria, especially in gut bacteria, remains an open question. Excessive amounts of cysteine are connected with oxidative stress, sulfide production, and overall growth inhibition (Dave and Shah 1997, Korshunov et al. 2020). In pathogenic bacteria such as *Neisseria meningitidis*, the depletion of cysteine from growth media led to a decrease in biomass of the pathogen (Waterbeemd et al. 2013). Lastly, depleting cysteine from the nutrition of mice can lead to a depletion of the internal CoA pool in murine cells, which is not rescued through bacterial cysteine production (Varghese et al. 2025). Potentially, depleting cysteine from bacteria might have a similar effect of the internal CoA pool; however, bacteria are commonly able to synthesize their own cysteine (Fujishima et al. 2018), making them less reliant on external cysteine sources.

Many gut bacteria synthesize cysteine from sulfides and O-acetyl-serine, which then serves as a sulfur source for other metabolites (Fig. 3) (Florin 1991, Seiflein and Lawrence 2001). Following its synthesis and consumption, bacterial genera, including *Streptococcus, Enterobacter, Fusobacterium, Escherichia, Desulfovibrio*, and *Clostridium* break down cysteine via cysteine desulfhydrase (EC 4.4.1.15), generating ammonia, pyruvate, and sulfide (Fig. 3) (Guarneros and Ortega 1970, Barton et al. 2017, Blachier et al. 2019).

The production of hydrogen sulfide (H_2_S) by the gut microbiota from cysteine, which is not used for CoA biosynthesis, is a common reaction and luminal sulfide concentrations can reach 0.3–3.4 mM (Suarez et al. 1997, Braccia et al. 2021). The impact of hydrogen sulphide on intestinal health is still debated; while previous studies suggested toxic effects on intestinal cells and gut bacteria (Christl et al. 1996, Attene-Ramos et al. 2010, Caffrey and Voordouw 2010), recent studies emphasize the positive impact on intestinal barrier function and bacterial survival (Mironov et al. 2017, He et al. 2025). Generally, the bacterial production of H_2_S is not damaging to intestinal cells, when the barrier function is intact and cells have intact detoxification systems (Furne et al. 2001). However, bacteria producing H_2_S have been correlated with inflammatory diseases and cancer (Birg and Lin 2025). This indicates that under homeostatic conditions the production of H_2_S is beneficial for both host and bacteria, while during inflammation, H_2_S and especially species producing H_2_S might worsen the phenotype. Therefore, a metabolic balance between CoA and H_2_S production from cysteine might be crucial to maintain gut homeostasis.

Apart from cysteine, sulfur is also obtained from taurine. Even though the direct impact of taurine on CoA metabolism in gut bacteria remains to be elucidated, taurine exerts various effects on the gut microbial metabolism. Taurine is synthesized from the cysteine-related metabolite cysteamine and contributes to taurine formation via hypotaurine in animal tissues (Fig. 3) (Cavallini et al. 1966). It is secreted by colonocytes and is abundantly present in the intestine, known to exert anti-inflammatory effects (Ahlman et al. 1993, Sasaki et al. 2017, Barbiera et al. 2022, Jornada et al. 2024).

Sasaki et al. (2017) investigated the impact of dietary taurine on the human colonic microbiota and found that most taurine remained undegraded under anaerobic conditions, whereas 83% was degraded in the presence of oxygen. Taurine did not significantly impact bacterial diversity, composition, or SCFA production suggesting its anti-inflammatory effects may stem from direct interactions rather than being mediated by bacterial metabolites (Sasaki et al. 2017).

Conversely, in mice, Qian et al. (2023) demonstrated significant alterations in intestinal microbiota upon taurine supplementations (Qian et al. 2023). This included increased abundance of the *Actinomycetota* and *Pseudomonadota* phyla, as well as the genera *Bifidobacterium* and *Bilophila*. Taurine supplementation also reduced total bile acid content, while enriching taurine-conjugated bile acids, suggesting distinct metabolic effects in murine systems compared to humans. These findings highlight that the impact of taurine on gut microbiota depends on both host species and environmental factors.

On a molecular level, *E. coli* e.g., expresses taurine utilization genes only under sulfur-limited conditions, indicating a back-up mechanism for sulfur acquisition (Eichhorn et al. 2000). Additionally, *Bilophila wadsworthia* is the only known gut bacterium capable of degrading taurine into sulfate, a metabolic process unique to this anaerobe (Laue et al. 1997, Laue et al. 2001). Recent studies showed that the presence and metabolic output of *B. wadsworthia*, which was mostly H_2_S and ethanol, can impact gut microbial communities and their metabolic capacity, linking the outcome of these interactions with cellular inflammation and increased gut permeability (Burkhardt et al. 2021, Sayavedra et al. 2025).

Thus, taurine acts as a homeostatic amino acid in the gut lumen, exerting varying effects on the microbiota of mice and humans. While its role in bacterial metabolism appears limited under normal gut conditions, taurine nonetheless influences microbial and host metabolic processes, underscoring its importance in gut health. Additionally, cysteine metabolism across bacterial species is tightly regulated, which underscores the significant role cysteine plays in shaping microbial interactions and metabolic pathways within the gut. Therefore, cysteine not only contributes to essential biosynthetic processes but may also influences bacterial community dynamics in the gut ecosystem, potentially being an essential factor in the balance between CoA and H_2_S production.

In summary, while pantothenate is well studied in the context of CoA biosynthesis, less is known about downstream intermediates such as 4′-phosphopantothenate and 4′-phosphopantetheine, particularly regarding their impact on bacterial communities. The sulfur-containing amino acid cysteine plays a central role in CoA biosynthesis and microbial metabolism, balancing between beneficial metabolic functions and potentially harmful H₂S production. In contrast, taurine, derived from cysteine, has a more context-dependent role, with limited use by gut microbes under normal conditions but notable immunomodulatory effects and influence on microbial composition under specific circumstances. Together, these metabolites shape microbial ecology and host interactions, highlighting the importance of metabolic balance between CoA biosynthesis and sulphur handling in maintaining gut homeostasis.

### The impact of CoA metabolites and derivatives as oral supplements and treatments

CoA metabolites have a great impact on all cellular life forms, which offers opportunities to use these metabolites or derivatives to aid metabolic diseases or infections. However, when evaluating the effect of oral treatment and supplementation on us, it is important to consider the interaction with the gut microbiota and how they convert and react to these compounds (Mousa et al. 2023).

The cysteine derivative N-acetylcysteine (NAC) is commonly used as anti-inflammatory drug and anti-oxidant (Tenório et al. 2021). However, its impact on gut function has only been recently elucidated in a mice model. The bacteria present in the murine gut impacted the permeability of NAC, which the authors suggested to be caused by a shift in sphingolipid synthesis in *Bacteroides thetaiotaomicron* and *Bacteroides fragilis*. The compound was hypothesized to be degraded into cysteine, which was then used to create palmitoyl-CoA, altering the bioavailability of NAC (Zhang et al. 2025). This indicates that derivatives of CoA intermediates can alter gut microbial metabolism and may be metabolized in a way that modifies their impact on the host.

Furthermore, analogues and derivatives of CoA intermediates have acquired significant interest as growth inhibitors and potential antimicrobial agents (Gerdes et al. 2002, Spry et al. 2008, Butman et al. 2020). Among these, N-substituted pantothenamides, which are pantothenate analogues, have been shown to inhibit the growth of lactic acid bacteria, including *Lactiplantibacillus plantarum, Lacticaseibacillus casei*, and *Lapidilactobacillus dextrinicus*, as well as *E. coli* (Clifton et al. 1970). De Villiers et al. (2014) explored the interactions between N-pentyl pantothenamide and different PanK types. For PanK-I in *E. coli*, the enzyme displayed similar activity with either pantothenate or N-pentyl pantothenamide suggesting that the analogue is converted into toxic intermediates during later steps in the pathway. In contrast, PanK-II from *S. aureus* exhibited a more complex response: enzyme inhibition at high concentrations and a stimulatory effect at lower concentrations (de Villiers et al. 2014). Pantetheine showed no inhibitory effect on PanK-I but it reduced PanK-II activity at high concentrations, possibly implicating its role in regulating PanK-II, which lacks the CoA-feedback inhibition characteristic of PanK-I (de Villiers et al. 2014). This might be especially relevant in studies that consider pantetheine, or its oxidized form pantethine, as oral supplement to aid disease since the effect on bacteria and how they metabolize it might be relevant to consider (Rumberger et al. 2011, Chen et al. 2023).

These findings suggest that N-substituted pantothenamides could be developed as selective antimicrobial agents. However, a key limitation arises from their degradation in serum by pantetheinases (Jansen et al. 2013). When combined with pantetheinase inhibitors, these analogues demonstrated enhanced antimicrobial effects, particularly against Gram-positive bacteria, such as *S. aureus* (Jansen et al. 2013). This underscores the potential for combining pantothenamide derivatives with other agents to overcome enzymatic degradation and enhance antimicrobial efficacy.

In contrast, in an earlier study, Pierpoint et al. (1955) investigated the influence of pantothenate derivatives on CoA biosynthesis and their growth promoting properties (Pierpoint et al. 1955). The authors demonstrated that pantothenylglycine exhibited growth effects comparable to pantothenate, promoting the growth of *L. plantarum* by 80%–100% relative to pantothenate. However, in *Morganella morganii*, only pantothenyl-β-alanine displayed growth-promoting properties, but achieving only 20%–30% of the growth observed with pantothenate (Pierpoint et al. 1955). These results suggest metabolic variations across bacterial species in CoA biosynthesis, which leads to the preferential metabolism of specific derivatives. Understanding how CoA derivatives are metabolized by different gut microbes, and how they shape community composition and function, is essential for their development as effective oral therapeutics.

### CoA metabolism and its metabolites in microbiota-related diseases

As established above, CoA metabolism and its associated metabolites are intricately tied to both mammalian and bacterial metabolic processes. Consequently, it is not unsurprising that various microbiota-related disease such as IBD and PD, have been linked to disruptions in CoA metabolism, as well as deficiencies or excess of specific metabolites (Cirstea et al. 2020, Shen et al. 2021).

In addition to these conditions, metabolic disorders, such as type 2 diabetes mellitus (T2D) and cardiovascular diseases have also been associated with compositional and functional changes in microbiota. These alterations suggest that CoA metabolism may play a pivotal role in the pathophysiology of these diseases, influencing both host and microbial metabolic networks (Qin et al. 2012, Cui et al. 2018).

### Inflammatory bowel disease

IBD is a chronic condition characterized by persistent inflammation of the gastrointestinal tract, influenced by genetic predisposition, environmental factors, immunological disruptions, diet, and imbalance in the gut microbiome (Podolsky 2002, Qiu et al. 2022). Several studies have highlighted significant changes in metabolite production associated with IBD. A hallmark of IBD is a reduced microbiota diversity and a diminished abundance of SCFA-producing bacteria, such as *F. prausnitzii* and *Roseburia spp*., leading to lower SCFA levels (Machiels et al. 2014, Yang et al. 2021, Ning et al. 2023, Yamamura et al. 2023). SCFAs are fermentative end products, intricately linked with CoA metabolism, that fuel fatty acid metabolism and maintain gut health (Lloyd-Price et al. 2019, Wang et al. 2021).

A direct impairment of bacterial CoA metabolism connected to IBD development has not been determined; however, pantothenate and the SCFA-producers *F. prausnitzii* and *R. hominis* were depleted in the guts of people with IBD (PwIBD), while facultative anaerobic bacteria such as *E. coli* were enriched in the samples (Lloyd-Price et al. 2019, Ning et al. 2023). Moreover, Santoru et al. (2017) identified alterations in pantothenate, β-alanine and CoA metabolism in PwIBD, including reductions in pantothenate and 3-hydroxybutyrate and increases in alanine and β-alanine (Santoru et al. 2017). Pantothenate appears to be an essential factor in disease induction since the complete lack of it induces human-like ulcerative colitis in pigs (Roediger 2019). Lastly, cysteine and taurine were hypothesized to be less available during colitis due to the presence of metabolic pathways in *Enterobacteriaceae* that take part in the degradation of sulfur-containing compounds (Richie et al. 2023). Therefore, CoA precursors appear to be depleted as a result of shifts in gut microbiota composition and metabolic activity, and are primarily metabolized by bacterial taxa that thrive in inflammatory conditions.

Apart from the direct CoA metabolism, various metabolic pathways linked to CoA are altered in IBD. Metabolic analysis, including untargeted metabolomics with ultra-high performance liquid chromatography/mass spectrometry (UPLC-MS/MS) and liquid-chromatography mass-spectrometry (LC-MS), have revealed disruptions in lipid metabolism in IBD (Table 1). Franzosa et al. (2019)demonstrated an enrichment of sphingolipids in fecal samples of PwIBD as well as α-amino acids, while long-chain fatty acids and triacylglycerols were reduced (Franzosa et al. 2019). Notably, Heimerl et al. (2006) found diminished lipid metabolism in intestinal tissues of PwIBD (Heimerl et al. 2006). Lloyd-Price et al. (2019) analysed faecal, colon, and blood samples and observed decreased metabolic diversity, including reduced polyunsaturated fatty acid levels (Lloyd-Price et al. 2019). Lastly, Ning et al. (2023) demonstrated an increase in amino acids and a decrease in organic acids related to the TCA cycle, which might be associated with CoA availability (Ning et al. 2023).

**Table 1.** Diseases associated with changes in microbiota composition and functionality related to CoA metabolism and related pathways.

Disease	Bacterial composition	Metabolic pathways	Reference
Inflammatory bowel disease	**Genus** *Enterococcus* ↑ *Escherichia* ↑ *Klebsiella* ↑ *Pseudomonadota* ↑ *Streptococcus* ↑ **Species** *Escherichia coli* ↑ **Phyla** *Bacillota* ↓ **Genus** *Butyricicoccus* ↓ *Clostridium* ↓ *Faecalibacterium* ↓ *Roseburia* ↓ **Species** *Faecalibacterium prausnitzii* ↓ *Roseburia hominis* ↓	Alanine metabolism ↑ Sphingolipids ↑ α-amino acids ↑ SCFA biosynthesis ↓ Pantothenate metabolism ↓ Polyunsaturated fatty acids ↓ Long-chain fatty acids ↓ Triacylglycerols ↓ Organic acids related to the TCA cycle ↓	(Machiels et al. 2014, Santoru et al. 2017, Franzosa et al. 2018, Lloyd-Price et al. 2019, Yang et al. 2021, Ning et al. 2023)
Parkinson’s disease	**Genus** *Akkermansia* ↑ *Bifidobacterium* ↑ *Bilophila* ↑ *Desulfovibrio* ↑ *Escherichia* ↑ *Lactobacillus* ↑ **Genus** *Bacteroides* ↓ *Blautia* ↓ *Lachnospira* ↓ *Pateruellaceae* ↓ *Roseburia* ↓	Amino acid metabolism ↑ Fucose degradation ↑ Nucleic acid degradation ↑ Fatty acid metabolism ↓ Pantothenate metabolism ↓ SCFA production ↓	(Unger et al. 2016, Baldini et al. 2020, Cirstea et al. 2020, Vascellari et al. 2020, Murros et al. 2021)
Type 2 diabetes mellites	**Genus** *Dorea* ↑ *Streptococcus* ↑ *Sutterella* ↑ **Species** *Akkermansia muciniphila* ↑ *Bacteroides caccae* ↑ *Clostridium hathewayi* ↑ *Clostridium symbiosum* ↑ *Desulfovibrio sp.3_1_syn3* ↑ *Eggerthella lenta* ↑ *Eggerthella sp*. ↑ *Escherichia coli* ↑ *Thomasclavelia ramosa* ↑ *Streptococcus salivarius* ↑ **Genus** *Clostridium* ↓ **Species** *Agathobacter rectalis* ↓ *Akkermansia muciniphila* ↓ *Clostridiales sp. SS3/4* ↓ *Faecalibacterium prausnitzii* ↓ *Roseburia intestinalis* ↓ *Roseburia inulinivorans* ↓	Membrane transport of sugars ↑ Membrane transport of branched-chain amino acids ↑ Sulphate reduction ↑ Butyrate biosynthesis ↓ Cofactor/vitamin metabolism ↓	(Qin et al. 2012, Allin et al. 2018, Zhong et al. 2019)
Atherosclerotic cardiovascular diseases	**Family** *Enterobacteriaceae* ↑ **Genus** *Collinsella* ↑ *Escherichia* ↑ *Streptococcus* ↑ **Species** *Eggerthella lenta* ↑ *Escherichia coli* ↑ *Klebsiella aerogenes* ↑ *Klebsiella spp*. ↑ *Mediterraneibacter gnavus* ↑ **Genus** *Bacteroides* ↓ *Segatella* ↓ *Eubacterium* ↓ *Roseburia* ↓ **Species** *Alistipes shahii* ↓ *Bacteroides spp*. ↓ *Faecalibacterium prausnitzii* ↓ *Segatella copri* ↓ *Roseburia intestinalis* ↓	Fatty acid degradation ↑ Glycerolipid metabolism ↑ Cystine transporters ↑ Branched-chain amino acid transporters ↑ Biosynthesis of pantothenate ↓ Biosynthesis of thiamine and riboflavin ↓ Taurine transport systems ↓	(Karlsson et al. 2012, Jie et al. 2017, Sayols-Baixeras et al. 2023)
Chronic heart failure	**Species** *Mediterraneibacter gnavus* ↑ *Streptococcus sp*. ↑ *Veillonella sp*. ↑ **Species** *Faecalibacterium prausnitzii* *Oscillibacter sp*. ↓ *Sutterella wadsworthensis* ↓	Lipid metabolism ↑ Amino acid synthesis ↓ Amino acid transport ↓ Butyrate production ↓	(Cui et al. 2018)

Changes in bacterial composition in fecal samples are indicated by increased abundances (↑) or decreased abundances (↓). Relevant metabolic pathway changes are noted if detected in fecal samples.

Altogether, IBD is characterized by significant disruptions in CoA-related metabolite production and downstream pathways. The depletion of pantothenate and SCFA-producing bacteria, along with increased levels of facultative anaerobes and disruption of CoA-dependent pathways such as lipid metabolism and TCA cycle, suggests a systemic shift in microbial metabolic states under inflammatory conditions. These changes may contribute to disease progression and gut homeostasis disturbance.

### Parkinson’s disease

PD is a neurodegenerative disorder characterized by the accumulation of α-synuclein in the nervous system. It has been associated with disruptions in gut microbiota, which may contribute to disease progression (Fasano et al. 2015). Studies have shown that people with PD (PwPD) exhibit notable changes in their gut microbiota, including an increase in the abundance of *Akkermansia, Escherichia*, and *Bifidobacterium* species, while *Bacteroides, Blautia*, and *Lachnospira* are reduced (Table 1) (Unger et al. 2016, Vascellari et al. 2020).

Metabolite analysis of PwPD has revealed reduced pantothenate production, along with decreased levels of linoleic and oleic acid, suggesting a disruption in vitamin and fatty acid pathways (Table 1). Notably, changes in pantothenate production positively correlated with the abundance of bacteria such as *Lachnospira, Pseudobutyrivibrio*, and *Roseburia*, while a negative correlation was observed with *Bifidobacterium* (Vascellari et al. 2020). Furthermore, Unger et al. (2016) observed a reduction in SCFA production, particularly in acetate, butyrate, and propionate, in fecal samples from PwPD (Unger et al. 2016). Cirstea et al. (2020) identified an enrichment in microbiome pathways linked to nucleic acid and fucose degradation, as well as amino acid metabolism (Table 1) (Cirstea et al. 2020), suggesting a shift from carbohydrate to protein metabolism in the intestines of PwPD. Furthermore, PwPD have higher amounts of *Desulfovibrio* and *Bilophila* present in their feces, which commonly produce H_2_S, potentially linked to disease progression and shifts in cysteine availability (Baldini et al. 2020, Murros et al. 2021, Buret et al. 2022). Conversely, Vascellari et al. (2020) reported an increase in ethanolamine, a glycerophospholipid intermediate linked with anti-inflammatory properties and cell proliferation, abundant in the gut (Zhou et al. 2017, Vascellari et al. 2020).

In conclusion, alterations in the gut microbiome and CoA-related metabolic pathways are significant in PwPD. These changes may impact the progression of the disease, highlighting the potential role of these microbes and their altered CoA metabolism in influencing PD pathology.

### Metabolic disorders and changes in microbiota composition and metabolism

Metabolic disorders, such as type 2 diabetes mellitus (T2D) and cardiometabolic diseases (e.g. atherosclerotic cardiovascular diseases and chronic heart failure), have become increasingly prevalent and are now associated with significant changes in gut microbiota composition and metabolic pathways. Research has highlighted the role of gut microbiota in influencing metabolic states and disease phenotypes, underlining the impact of these microbiota alterations on disease progression (Fan and Pedersen 2021).

#### Type 2 diabetes mellitus

T2D has been linked to gut microbiota alterations. Fecal microbiota from individuals with T2D show a reduction in butyrate-producing species including *A. rectalis, F. prausnitzii*, and *R. intestinalis*, with an enrichment of opportunistic pathogens such as *E. coli* and *Thomasclavelia ramosa* (Table 1) (Qin et al. 2012). Functional changes, as indicated by metagenomic analysis, reveal an enrichment of genes related to sugar and branched-chain amino acid transport, while butyrate, cofactor, and vitamin biosynthesis pathways were diminished (Qin et al. 2012). These results suggest that T2D may be associated with a pathogen-dominant microbiota and decreased production of metabolites such as butyrate and vitamins. While CoA was not directly investigated, the altered production of metabolites downstream of CoA biosynthesis, such as butyrate, might hint a potential disruption in the pathway since butyrate production relies on the presence of CoA (Flint et al. 2015).

Research comparing T2D and prediabetes, a condition characterized by elevated blood glucose levels, has shown mixed results regarding gut microbiota composition. While *A. muciniphila* was found to be enriched in individuals with T2D, Allin et al. (2018) observed a decrease in its abundance in individuals with prediabetes (Qin et al. 2012, Allin et al. 2018). This discrepancy may be due to the impact of drugs such as metformin, commonly used to treat T2D, which can alter gut microbiota independently of disease status (Forslund et al. 2015, Allin et al. 2018). This highlights the importance of distinguishing between drug-induced and disease-related microbiota changes.

Next to *A. muciniphila*, prediabetic individuals exhibited increased abundance of *Dorea, Sutterella*, and *Streptococcus* (Allin et al. 2018). Zhong et al. (2019) investigated fecal samples of individuals with prediabetes in a Chinese cohort and found lower abundance of *F. prausnitzii* compared to healthy and treated controls, while *E. coli* and and *Eggerthella sp*. were increased in pre-diabetic individuals (Zhong et al. 2019). While *F. prausnitzii* is a known butyrate producer (Lopez-Siles et al. 2017) and relies on the presence of external pantothenate, *E. coli* can produce its own pantothenate (Magnúsdóttir et al. 2015). Furthermore, *Eggerthella sp*. uses acetate a carbon source and also expresses pantothenate biosynthetic genes, potentially providing a growth benefit (Noecker et al. 2023). Shifts in the gut ecosystem, particularly in the availability of substrates such as pantothenate, may create a beneficial environment for these species, while species such as *F. prausnitzii* fall behind, possibly inducing a vicious cycle of dysbiosis.

#### Atherosclerotic cardiovascular diseases and chronic heart failure

*Atherosclerotic cardiovascular diseases* (ACVD), a disease associated with inflammation and dysfunction in the heart and vascular system, has been connected with changes in the gut microbiota as well (Ellulu et al. 2016). Jie et al. (2017) found an enrichment of *Streptococcus, Enterobacteriaceae, E. lenta*, and *M. gnavus* in the gut microbiota of individuals with ACVD, while taxa such as *Bacteroides spp., R. intestinalis, F. prausnitzii, Segatella copri*, and *Alistipes shahii* were reduced (Table 1) (Jie et al. 2017). Furthermore Sayols-Baixeras et al. (2023) confirmed the higher abundance of *Streptococcus* in individuals with atherosclerosis (Sayols-Baixeras et al. 2023). Metagenomic analysis revealed a decreased ability for biosynthesis of vitamins, including pantothenate, and an increase in pathways for fatty acid degradation and glycerolipid metabolism. Notably, CoA metabolism was slightly enriched in ACVD patients, and taurine transporters were reduced (Table 1) (Jie et al. 2017). Similarly, *Collinsella* was found to be enriched in individuals with atherosclerosis, while the fibre degraders *Bacteroides spp*. and butyrate-producers *Eubacterium* and *Roseburia* were less abundant (Karlsson et al. 2012).

Chronic heart failure, often a consequence of cardiovascular diseases, also shows significant alterations in gut microbiota. Cui et al. (2018) identified an enrichment in *M. gnavus, Streptococcocus sp*., and *Veillonella sp*., while SCFA producers such as *F. prausnitzii, Oscillibacter sp*., and *Sutterella wadsworthensis* were reduced (Table 1). Kyoto Encyclopedia of Genes and Genomes (KEGG) pathway analysis revealed a decrease in amino acid synthesis and butyrate production, alongside an increase in lipid metabolism genes (Cui et al. 2018), reflecting an inflammatory microbiota phenotype in CHF.

Overall, research on metabolic disorders has consistently demonstrated shifts in gut microbiota composition, with a trend towards a reduction in health-promoting species (e.g. SCFA producers) and an enrichment in opportunistic pathogens. These microbiota changes are accompanied by functional alterations in metabolic pathways, including reduced butyrate, CoA and vitamin biosynthesis, including pantothenate, and an increase in lipid and branched-chain amino acid metabolism. Given the central role of CoA in fatty acid pathways and cellular energy production, its dysregulation may contribute to the dysbiosis seen in gut microbiota in individuals with metabolic disorders. Collectively, these findings highlight a common phenotype in metabolic disorders, where changes in microbiota composition and functionality are linked to metabolic abnormalities and disease progression (Table 1).

### Experimental settings to study CoA metabolism in gut bacteria

To fully understand CoA metabolism within the gut microbiome, various assays and experimental models are required. These methods encompass biochemical assays, genetic tools, and omics approaches, as well as the use of model systems for *in vivo* and *in vitro* studies. This section reviews current methodologies for investigating CoA metabolism, emphasizing experimental approaches that enhance our ability to study its dynamics and impact.

#### Biochemical, microbiological, and enzymatic assays

Enzymatic and metabolic assays have long been utilized to analyse metabolic fluctuations. For example, Jackowski and Alix (1990) developed a pantothenate transport assay using [1–^14^C]-labelled pantothenate and thin-layer chromatography to assess the activity of the pantothenate permease PanF (Jackowski and Alix 1990). Similar labelling can also be applied for other metabolites involved in CoA metabolism, such as CoA itself and ACP. For example, Jackowski and Rock (1984) applied [^3^H]-labelling to track these molecules (Jackowski and Rock 1984). Additionally, Mercer et al. (2009) employed metabolic labelling of carrier proteins by incorporating pantetheine analogues into CoA, which are amenable to labelling. This approach enabled the visualization and identification of fatty acid ACPs from various bacterial species (Mercer et al. 2009). Furthermore, 4′-phosphopantetheine and pantetheine can also be isotopically labelled, and their metabolism can be traced in multiple microbial species (Yu et al. 2022). Lastly, Deng et al. (2021) developed an untargeted stable isotope-resolved metabolomics technique to trace the fate of dietary fibres labelled with ^13^C, which shows their incorporation in pantothenate and CoA metabolites and might be applied to CoA metabolites as well (Deng et al. 2021). The labelling was followed by either protein or metabolite extraction and changes were determined either by thin-layer chromatography (Jackowski and Rock 1984, Jackowski and Alix 1990) or mass spectrometry and LC-MS (Mercer et al. 2009, Yu et al. 2022).

Beyond labelling, enzyme activity and metabolite formation can be quantified through NADH formation in cell extracts and gas chromatography. Claesson et al. (1990), e.g. grew *Fusobacterium* cultures with volatile sulfur compounds such as cysteine and methionine, then extracted and analysed the culture vapour for gas production using gas chromatography, while assessing enzyme activity via NADH measurements in cell extracts (Claesson et al. 1990). Earlier studies show that pantetheine can be determined via different enzymatic assays that detect the hydrolysis products such as cysteamine and pantothenate (Wittwer et al. 1982). Lastly, an indirect enzyme linked immunosorbent assay has been employed to measure pantothenate in plasma samples, which might be translatable to bacterial supernatant (Song et al. 1990).

In addition to enzymatic assays, pantothenate production has previously been assessed by evaluating the growth of *Lactobacillus plantarum*, a pantothenate auxotroph, based on the principle that its growth is proportional to pantothenate concentrations (Strong et al. 1941). Thus, a variety of methods exists to evaluate metabolite production, enzyme activity, and metabolic flux, all of which offer robust tools for studying CoA metabolism.

While these methods were employed earlier to study CoA metabolism and are all based on cell lysates, evaluating only moments in the metabolism, it is of great value to understand metabolic fluxes in live cells. Smith et al. (2025) developed a biosensor with high selectivity towards acetyl-CoA over CoA and its other thioesters and proved that fluctuations can be readily detected (Smith et al. 2025). Lastly, click chemistry might be applied in the future to label CoA metabolites or enzyme to trace and evaluate their flux in bacterial cells, which is done by labelling metabolites with a small chemical group that can bind to a detectable compound (van Kasteren and Rozen 2023). Similar approaches and methods might be employed in the future to better understand the flux and balances of CoA metabolites in bacterial cells.

#### Genetic engineering approaches

Genetic engineering of CoA-related enzymes offers valuable insights into their roles within bacterial metabolism (Ku et al. 2020). While much of the focus in genetic engineering has been on enhancing the production of specific metabolites, such as acetyl-CoA, similar strategies could be applied to enzymes involved in CoA biosynthesis (Zhang et al. 2019, Ku et al. 2020). For example, Wang et al. (2024) recently engineered the pantothenate pathway in *E. coli* to overproduce the vitamin (Wang et al. 2024). Additionally, disrupting known enzymes of the pantothenate pathway using knock-out systems can help redirect metabolic fluxes, thereby mimicking disease states characterized by enzyme deficiencies or dysfunctions.

Such approaches may enable researchers to investigate the downstream effects of these metabolic alterations on cellular physiology and microbial communities.

#### Metabolite extraction and quantification

Commonly, metabolites are directly measured after extraction from bacterial cells. Techniques such as thin-layer chromatography (Jackowski and Alix 1990) and both targeted and untargeted metabolomics provide valuable insights into metabolite production of bacterial communities (Rooks and Garrett 2016). For example, Seifar et al. (2013) developed a quantitative method for assessing intracellular CoA and its thioesters using ion pair reversed phase ultrahigh-performance liquid chromatography coupled with tandem mass spectrometry (Seifar et al. 2013). Similarly, Shimazu et al. (2004) quantified intracellular CoA by adapting silicone oil layer centrifugation for bacterial applications and analysing the extracted compounds using reversed-phase high-performance liquid chromatography (Shimazu et al. 2004). Additionally, Gläser et al. (2020) employed a method to quantify CoA and its thioesters with ^13^C-labelling and Mass Isotopomer Ratio Analysis, originally developed by Mashego et al. (2004) (Mashego et al. 2004, Gläser et al. 2020). The total CoA content in cells can also be measured by derivatizing CoA and its thioesters, which can then be detected in a high-performance liquid chromatograph with either an absorbance or fluorescence detector (Frank et al. 2019). Furthermore, pantothenate is commonly detected in various samples with LC-MS (Mittermayr et al. 2004).

It is also possible to combine the detection of multiple CoA metabolites into one method, which was demonstrated by Jones et al. (2021). They developed a method to simultaneously detect pantothenate, dephospho-CoA, CoA, and downstream CoA metabolites such as acetyl-CoA, malonyl-CoA,and succinyl-CoA in biological samples with LC-MS/MS (Jones et al. 2021).

While *in vitro* assays for studying CoA metabolism have long been established, the integration of advanced methodologies such as metabolomics has greatly improved the ability to explore CoA metabolism in greater depth. Indeed, the advent of metabolomics has significantly enhanced gut microbiome studies, enabling comprehensive analyses of changes in metabolite production (Jarmusch et al. 2020, Deek et al. 2024).

#### In vivo approaches

While *in vitro* assays are invaluable for understanding specific biochemical mechanisms, *in vivo* experiments are crucial for capturing the dynamic nature of metabolic flux within an organism. Direct analysis of metabolic flux in humans can be performed using labelled metabolites, which allows for the visualization of tracers after extraction. For example, Radziuk and Pye (2001) investigated glucose metabolic flux in people with diabetes mellitus by infusing [6–^3^H]-glucose and measuring plasma concentrations over a 10-h period (Radziuk and Pye 2001). This method effectively tracked glucose metabolism *in vivo*. Similarly, stable-isotope tracers have been widely employed in other studies to assess metabolic flux in various contexts (Wolfe and Peters 1987, Wolfe et al. 1987, Bednarski et al. 2021, Yu et al. 2022). Xue et al. (2023) developed a biosensor for CoA measurements in living human cells by labelling proteins with synthetic fluorescent probes, which enabled them to investigate CoA homeostasis and transport mechanisms in cells (Xue et al. 2023). In the future, it might be feasible to translate this tool to bacterial cells to analyse their CoA metabolism. These methodologies demonstrate the utility of *in vivo* experiments for providing a comprehensive view of metabolic flux, complementing insights gained from *in vitro* studies.

In addition to studying metabolic flux, mouse models have been widely used to investigate the effects of specific metabolites, such as taurine, on the gut microbiota. For example, Qian et al. (2023) analysed the compositional and metabolic changes within the microbiome community following taurine ingestion to better understand its impact (Qian et al. 2023). This study employed metagenomics for microbiome profiling, LC-MS for bile acid measurements, and enzyme-linked immunosorbent assays for cytokine quantification in the murine colon (Qian et al. 2023). The combination of these methods provided a comprehensive understanding of the effect of taurine on the gut microbiome.

Beyond taurine, hopantenate, structural analogue of pantothenate, was tested as a chemical inhibitor of PanKs by administering it in mice. Physiological effects were particularly evident in the liver, affecting lipid, protein, and organic acid metabolism (Zhang et al. 2007). However, Mostert et al. (2021) demonstrated that hopantenate is a substrate for PanK and phospho-hopantenate is a chemical inhibitor of the downstream enzyme PPCS (Mostert et al. 2021). These findings suggest that physiological effects were due to inhibition of PPCS, impairing CoA biosynthesis. Furthermore, Zhang et al. (2007) observed that hopantenate is not a substrate for *E. coli* PanK-I, indicating that the influence of hopantenate may be specific to eukaryotic enzymes (Zhang et al. 2007). Comparable approaches to target the gut microbiota could be adapted to assess the effects of other inhibitors on CoA metabolism. Collectively, *in vivo* experiments in both human and animal models offer versatile platforms to study the systemic and localized impacts of alterations in CoA metabolism, enhancing our understanding of these pathways in health and disease.

### Concluding remarks

CoA metabolism is essential for all living organisms and plays a central role in key metabolic pathways, including fatty acid metabolism and TCA cycle that underpin bacterial growth and community dynamics (Jackowski and Rock 1981, Leonardi and Jackowski 2007). Investigating CoA metabolites and their impact on gut bacterial communities is therefore critical for understanding how shifts in bacterial metabolism influence gut health and disease states driven by microbiota and metabolic changes.

In this review, we demonstrated that while CoA biosynthesis is highly conserved across bacteria, its regulation varies and pantothenate biosynthesis, the precursor to CoA, is species-dependent with some bacteria relying on transporter expression (Jackowski and Alix 1990, Zhang et al. 2014, Ernst and Downs 2015). Intermediates and derivatives of the CoA pathway modulate bacterial growth and enzyme expression, shaping microbial communities under different metabolic conditions, such as inflammation or homeostasis (Balibar et al. 2011, de Villiers et al. 2014). These metabolic shifts are especially relevant in the context of disease, where altered CoA metabolism corresponds with changes in bacterial composition and functionality, contributing to inflammatory or pathogenic profiles (Cui et al. 2018, Lloyd-Price et al. 2019, Pereira et al. 2022).

Despite decades of research, key gaps remain, such as identifying transport mechanisms for pantothenate and phosphate-containing intermediates, which have yet to be characterized (Jackowski and Rock 1984, Gerdes et al. 2002, Leonardi and Jackowski 2007), and clarifying widespread distribution of PanK-III in gut bacteria (Vallari et al. 1987, Yang et al. 2006, Zhang et al. 2014, Shimosaka et al. 2016). Furthermore, metabolites such as pantethine and pantetheine show species-specific effects on bacterial growth but their uptake pathways are poorly understood with only cysteinopantetheine uptake demonstrated via a cystine transporter in *B. subtilis* (Balibar et al. 2011, de Villiers et al. 2014, Ernst and Downs 2015, Warneke et al. 2024).

Given the central role of CoA in metabolic networks, its dysregulation can directly affect bacterial fatty acid metabolism, the TCA cycle, pathways crucial to microbial production of health-relevant metabolites, such as SCFAs, and to maintaining gut barrier integrity (Unger et al. 2016, Cui et al. 2018, Cirstea et al. 2020). This underscores the importance of connecting CoA metabolism more explicitly to gut microbial function and disease processes.

Current microbiome studies largely rely on fecal samples from limited geographical cohorts, which do not fully represent gut microbial diversity or function (Qin et al. 2012, Allin et al. 2018). Broader sampling methods, such as pH-sensitive capsules, and studies across diverse populations are needed to better understand how CoA metabolism influences microbial communities and human health (Nejati et al. 2022, Shalon et al. 2023, Procházková et al. 2024). Additionally, drug treatments can confound microbiota composition, irrespective of the disease state, highlighting the need for validated models (Forslund et al. 2015). In summary, CoA metabolism is a pivotal hub linking bacterial physiology to gut ecosystem function and disease. Addressing these remaining knowledge gaps will advance the understanding of microbiota-related pathologies and may reveal novel therapeutic targets.

## Data Availability

The data underlying this article are available in the National Center for Biotechnology Information at https://www.ncbi.nlm.nih.gov/nucleotide/, accession numbers of the sequences can be found at Yang et al. (2006).
